# 4027 Collaborative Working Retreats for Interdisciplinary Investigators and Engaged Stakeholders as a Tool for Sparking Creativity and Accelerating the Development of Translational Research Projects

**DOI:** 10.1017/cts.2020.354

**Published:** 2020-07-29

**Authors:** Elaine A Borawski, Anna Thornton Matos, Meredith Goodwin, Rachel Ann Gardenhire, Briana McIntosh

**Affiliations:** 1 Case Western Reserve University

## Abstract

OBJECTIVES/GOALS: As part of the Cleveland CTSA, “Collaborative Working Retreats” have been developed for the purpose of being a catalyst to move groups of interdisciplinary investigators and stakeholders to collaborative research teams with feasible and actionable translational research projects. METHODS/STUDY POPULATION: Groups of interdisciplinary investigators with engaged stakeholder(s) were invited to apply. Selected groups participated in a 4-hour, professionally facilitated retreat, tailored to the unique needs of each team. In addition to the facilitator, a graphic recorder was utilized to capture ideas and aid in decision making by creating a visual narrative linked to the team’s overall vision. Teams were charged with generating three translational research projects and writing a formal Team Action Plan (TAP) by two months post retreat. Retreat participants were asked to complete a survey to evaluate the retreat, and structured interviews were conducted with team leaders 4-6 months post retreat. RESULTS/ANTICIPATED RESULTS: Six groups were awarded retreats, comprised of 48 investigators (representing all schools in the university and 3 of 4 affiliated hospital systems) and 28 stakeholders for a total of 76 participants. 45% completed the followup survey. 77% said they would recommend the service to other teams or would use it again themselves and 97% stated their team benefited from having a facilitator. At 2 month follow up, one team had completed the TAP and subsequently applied for federal funding. However, 4 of the remaining 5 teams indicated that they had made significant progress, attributing progress to their retreat time. Each teams’ progress is being tracked for 2 years, using a newly developed metric. DISCUSSION/SIGNIFICANCE OF IMPACT: Facilitated retreats appear to serve as an important catalyst for progression of translational research projects, providing needed time and support for brainstorming and planning. Lessons learned, pre-retreat work, and tools for tailoring retreat content and tracking progress will be presented.

